# Validity of upfront surgery for patients with unsuspected lymph node metastasis in esophageal cancer: a propensity scoring matching study

**DOI:** 10.1186/s13019-018-0757-y

**Published:** 2018-06-07

**Authors:** Jae Kil Park, Jae Jun Kim, Seok Whan Moon, Deog Gon Cho

**Affiliations:** 1 Department of Thoracic and Cardiovascular Surgery, Seoul St. Mary’s Hospital, The Catholic University of Korea College of Medicine, Seoul, South Korea; 2 Department of Thoracic and Cardiovascular Surgery, Uijeongbu St. Mary’s Hospital, The Catholic University of Korea College of Medicine, 271 Cheonbo Street, Uijeongbu City, Gyeonggi-do 480-717 South Korea; 3 Department of Thoracic and Cardiovascular Surgery, St. Vincent’s Hospital, The Catholic University of Korea College of Medicine, Suwon, South Korea

**Keywords:** Esophageal cancer, Lymph node metastasis, Neoadjuvant therapy

## Abstract

**Background:**

Although neoadjuvant therapy followed by esophagectomy is well-established as being superior to upfront esophagectomy when locoregional lymph node (LN) metastasis is present in esophageal cancer, upfront esophagectomy without neoadjuvant therapy may be performed in patients with LN metastasis due to unreliable preoperative evaluations. However, outcomes in this setting remain unclear. The purpose of the present study was to clarify whether upfront esophagectomy without neoadjuvant therapy in patients with unsuspected lymph node metastasis in esophageal cancer is appropriate.

**Methods:**

We included 215 squamous cell esophageal cancer patients who met the study criteria. Inclusion criteria included complete (R0) and curative surgery cases, intra-thoracic esophageal cancer, preoperative biopsy-proven squamous cell carcinoma, and cases without LN metastasis (WL, cN0 and pN0) or with unsuspected LN metastasis (UL, cN0 and pN1). Exclusion criteria were palliation or salvage cases, other uncured previous or current primary cancers, complete remission cases, and operative mortalities (defined as patients who died during hospitalization or within one month after surgery). We compared 5-year disease- free survival (DFS) between WL and UL. In addition, we investigated the influence of neoadjuvant therapy in UL. To overcome heterogeneity in baseline characteristics between the groups, a propensity matched-analysis based on propensity scores was then carried out to create a cohort of WL with clinical characteristics similar to those in UL.

**Results:**

The incidence of UL among preoperative N0 patients was 25.6% and the incidence of UL cases who did not receive neoadjuvant therapy was 47.2%. All subjects were stratified into either WL (160 patients) or UL (55 patients). Twenty nine of 55 patients in UL received neoadjuvant therapy before esophagectomy and all patients with LN metastasis received adjuvant therapy after esophagectomy. There was no significant difference in DFS between WL and UL (*p* = 0.242). Furthermore, there were no significant differences in DFS between cases that received and did not receive neoadjuvant therapy (*p* = 0.769).

**Conclusions:**

Upfront surgery without neoadjuvant therapy in UL is appropriate for patients who can tolerate adjuvant therapy.

## Background

The basic treatment strategy for esophageal cancer depends on the cancer stage [1–5]. Neoadjuvant therapy is usually recommended for patients with lymph node (LN) metastases in esophageal cancer [2, 4, 6]. In esophageal cancer, positron emission tomography computed tomography (PET-CT) is essential to the evaluation of the cancer stage, especially with respect to distant or locoregional LN metastases [7–10]. A lesion is usually regarded to be malignant or metastatic when the maximum standardized uptake value is ≥2.5 and the size is ≥1 cm on PET-CT and endoscopic ultrasound [11, 12]. However, discrepancies between the preoperative evaluations and the pathologic findings are not uncommon, especially in locoregional LN [12, 13]. Because thoracic surgeons do not usually perform an invasive evaluation for locoregional LN when the LN is negative on PET-CT and endoscopic ultrasound, the preoperative evaluation might provide inaccurate information and influence the management plan and prognosis. Although neoadjuvant therapy followed by esophagectomy is well-established as being superior to upfront esophagectomy when LN metastasis is present in esophageal cancer, there are uncertainties regarding upfront esophagectomy without neoadjuvant therapy for clinically unsuspected LN metastasis cases [7, 11, 14, 15]. In reality, due to unreliable preoperative clinical evaluation, upfront esophagectomy without neoadjuvant therapy may be performed in a patient with LN metastasis. However, outcomes in this setting remain unclear [16]. In other words, whether prognosis varies between patients without LN metastasis (WL: cN0 and pN0) and those with unsuspected LN metastasis (UL: cN0 and pN1) needs to be elucidated. Additionally, although complete resection is an important prognostic factor for esophageal cancer, it remains unclear whether neoadjuvant therapy is beneficial for UL. The purpose of the present study was to clarify whether upfront esophagectomy without neoadjuvant therapy is appropriate in UL.

## Methods

### Study subjects and methods

We retrospectively compiled and analyzed data from patients who had undergone curative and complete surgery for intra-thoracic esophageal cancers at a single tertiary Korean hospital from January 2009 to December 2015. Inclusion criteria were complete (R0) and curative surgery cases, intra-thoracic esophageal cancer, preoperative biopsy-proven squamous cell carcinoma, and cases with WL or UL. Exclusion criteria were palliation or salvage cases, other uncured previous or current primary cancers, complete remission cases after neoadjuvant therapy, and operative mortalities (defined as patients who died during hospitalization or within one month after surgery). The preoperative evaluations included esophagogastroduodenoscopy, esophagography, chest CT, abdominal CT, PET- CT, endoscopic ultrasound, and bone scan. In the present study, lesions were regarded as malignant or metastatic when the maximum standardized uptake value was ≥2.5 and the size was ≥1 cm on PET- CT and endoscopic ultrasound. Neoadjuvant and adjuvant therapies were usually performed following the National Comprehensive Cancer Network guidelines or the recommendations from a multidisciplinary team who assessed cancer status and each patient’s condition [4, 17]. Neoadjuvant therapy usually consisted of two cycles of cisplatin and 5-fluorouracil, plus 25 fractions of radiation therapy (over five weeks) to a total of 41–45 Gray. Re-evaluation by PET- CT was performed four weeks after completion of neoadjuvant therapy, and further management was determined. In those cases receiving neoadjuvant therapy, surgery was usually preformed five or six weeks after neoadjuvant therapy completion. In the present study, preoperative stage in neoadjuvant cases was defined as clinical stage by PET-CT reevaluation after completion of neoadjuvant therapy and before surgery. Surgeries were performed by two thoracic surgeons using Ivor Lewis or McKeown procedure depending on cancer status. Patients were usually followed at 3-month intervals for one year after treatment completion, including adjuvant therapy, and then at 6-month intervals. Recurrence or metastasis was diagnosed based on imaging findings including PET-CT, brain MRI, and bone scan, or pathological confirmation when clinically feasible. Cancer stage was determined with regard to the seventh American Joint Committee on Cancer staging system.

UL status is defined as having pathologic evidence of metastatic esophageal cancer in LN after esophagectomy in preoperative cases without LN metastasis (i.e. cN0 on the preoperative evaluations, but pN1 on the postoperative pathologic findings).Since most patients were in an early cancer stage, the sample size was small, and various factors after recurrence can influence overall survival, we carried out a 5 -year disease-free survival (DFS) study instead of an overall survival study to clarify whether upfront esophagectomy without neoadjuvant therapy in UL is appropriate. To clarify the validity of upfront esophagectomy in UL, we compared DFS between WL and UL. In addition, we investigated the benefit of neoadjuvant therapy in UL.

### Statistical considerations and study approval

The Student’s T test was used to compare the groups with continuous variables. The Chi-squared test or the Fisher’s exact test was used to evaluate the associations between the groups with categorical variables as appropriate. Disease-free intervals were measured from time of surgery to time of recurrence or last follow-up. DFS between WL and UL was compared using the Kaplan-Meier method with log-rank test. The influence of neoadjuvant therapy on prognosis in UL was also evaluated using the Kaplan-Meier method with log-rank test. To overcome heterogeneity in baseline characteristics between the groups, a matched-analysis based on propensity scores was then carried out to create a cohort of WL patients with clinical characteristics similar to those in the UL. Covariates included age, sex, pathological T stage, method of esophagectomy, and receipt of neoadjuvant therapy. All statistical analyses were performed using the Statistical Package of Social Sciences version 22.0 (Chicago, IL, USA). A p- value less than 0.05 (two- sided) was regarded as statistically significant. This study was approved by the Institutional Review Board of Seoul St. Mary’s Hospital (IRB approval number: KC17RESI0293).

## Results

### Study subjects

The incidence of UL among preoperative N0 patients was 25.6% and the incidence of UL cases who did not receive neoadjuvant therapy was 47.2%. We included 215 patients (male 199, female 19; mean age 62.8 ± 8.6 years) who met the study criteria. All subjects were stratified into either WL (160 patients) or UL (55 patients). Twenty nine of 55 patients in UL received neoadjuvant therapy before esophagectomy and all patients with LN metastasis received adjuvant therapy after esophagectomy. All cancer histologies confirmed squamous cell carcinomas. Tumors were located in the upper thoracic esophagus (25 cases), middle thoracic esophagus (109 cases), and lower thoracic esophagus (81 cases). The mean tumor length and size were 3.0 (±2.2) cm and 8.3 (±13.8) cm^2^, respectively. The mean number of LN dissected was 24.3 (±11.0). The mean observation period was 29.3 (±20.3) months. Recurrence was found in 46 patients during the observation period. The overall clinico-pathologic characteristics of the study subjects are summarized in Table 1.

**Table 1:** .Overall clinic-pathologic characteristics for the study subjects.

Variables		WL (*N* = 160)	UL(*N* = 55)	*P*- value
Age (year)		63.5(±9.1)	60.7 (±6.7)	0.014
Sex	Male	144	55	0.014
	Female	16	0	
Preoperative T stage	1	95	23	0.001
	2	50	11	
	3	15	21	
Postoperative T stage	1a	48	3	< 0.001
	1b	68	13	
	2	19	21	
	3	25	18	
Location of cancer	Upper thoracic	22	3	0.254
	Middle thoracic	78	31	
	Lower thoracic	60	21	
Method of surgery	Ivor Lewis	148	45	0.080
	McKeown	114	10	
Differentiation	Well	24	3	0.351
	Moderate	109	52	
	Poor	27	0	
Neoadjuvant therapy	No	136	26	< 0.001
	Yes	24	29	

### Prognosis between WL and UL

**Fig. 1:**
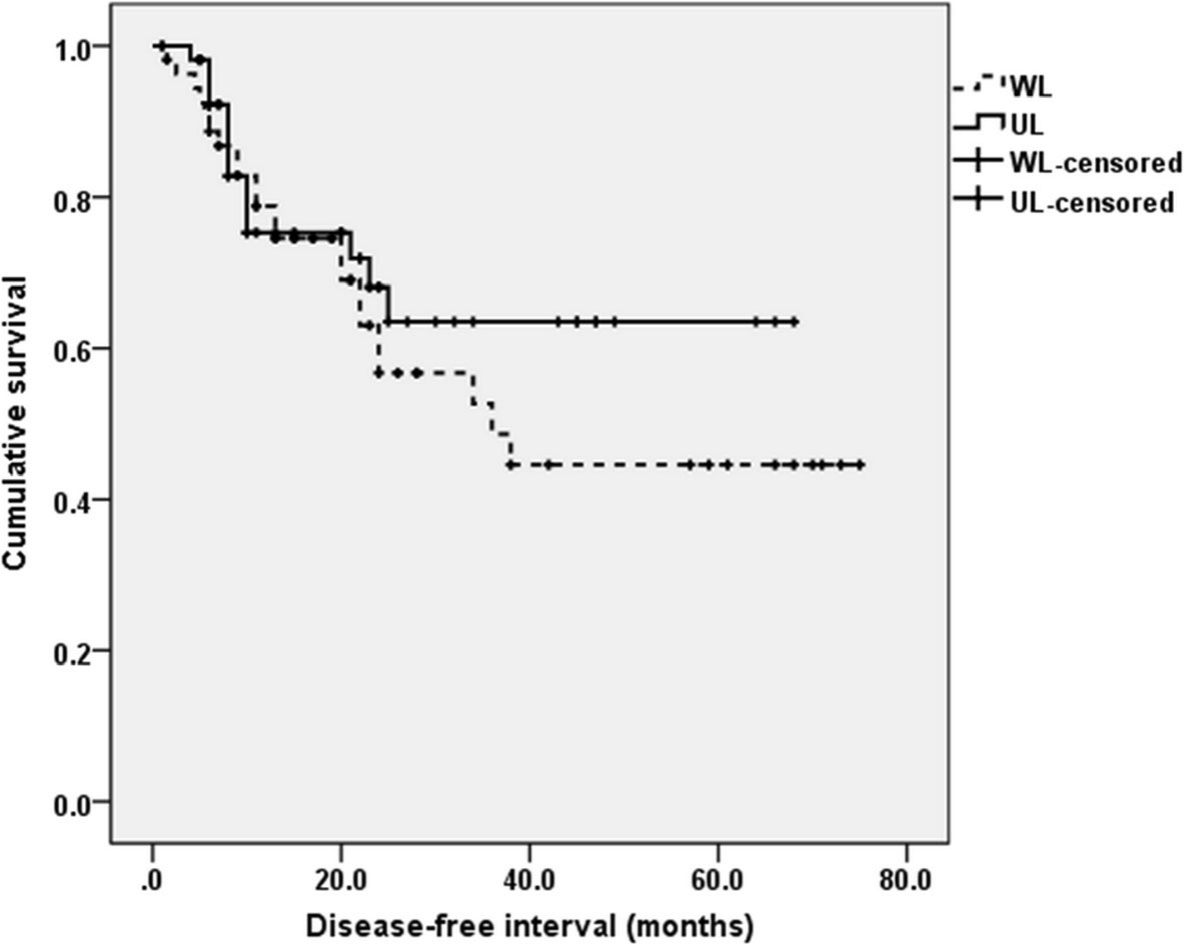
Prognosis between patients without lymph node metastasis and those with unsuspected lymph node metastasis. There was no significant difference in 5-year disease-free survival between patients without lymph node metastasis (WL) and those with unsuspected lymph node metastasis (UL) after propensity score matching (*p* = 0.345)

There were 160 WL and 55 UL patients. Fifty three patients received neoadjuvant therapy (24 in WL and 29 in UL). We analyzed DFS between WL and UL after propensity score matching to overcome heterogeneity between WL and UL. Covariates included age, sex, neoadjuvant therapy, method of surgery, and pathological T stage (Table 2).There were 55 WL cases and 55 UL cases after propensity score matching. There was no significant difference in DFS between WL and UL (Fig. 1. *p* = 0.242).

**Table 2:** Propensity score matching description

Covariates	Total Population				Propensity-matched Population			
	WL (n = 160)	UL (n = 55)	*p*-value	standardized difference	WL (*n* = 55)	UL (n = 55)	*p*-value	standardized difference
Age	63.5 ± 9.1	60.7 ± 6.7	0.014	−0.430	61.0 ± 9.4	60.7 ± 6.7	0.842	−0.046
Sex (Male)	144 (90%)	55 (100%)	0.014	N/A	55(100%)	55(100%)	N/A	N/A
NT	24(15%)	29(52.7%)	< 0.001	0.749	21(38.2%)	29(52.7%)	0.180	0.289
Ivro Lewis	146	45	0.080	0.242	42	45	0.640	−0.140
pT Stage	2.1 ± 1.0	3.0 ± 0.9	< 0.001	0.953	2.6 ± 1.1	3.0 ± 0.9	0.132	0.448

### Influence of neoadjuvant therapy in UL

**Fig. 2:**
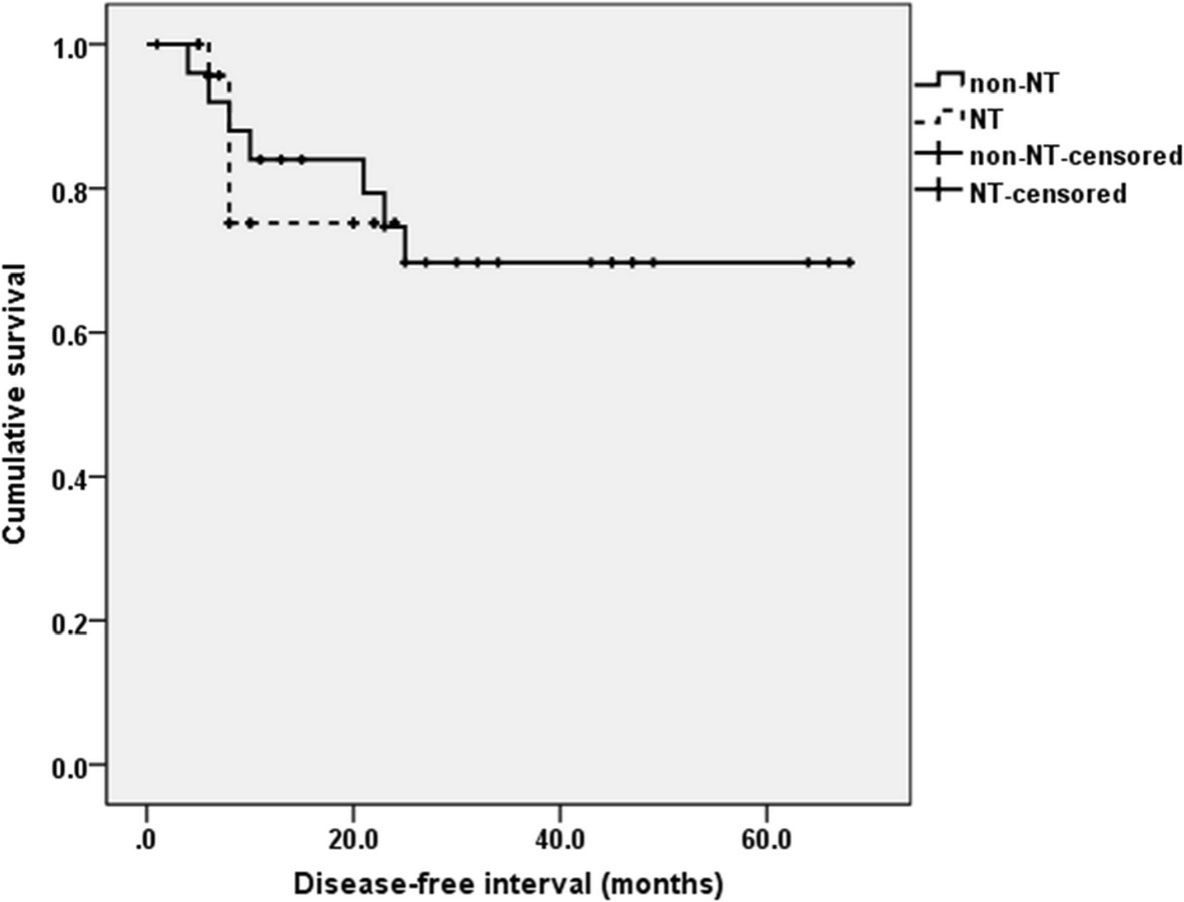
Influence of neoadjuvant therapy in patients with unsuspected lymph node metastasis. There was no significant difference in 5-year disease-free survival between patients who receive (NT) or did not receive neoadjuvant therapy (non-NT) after propensity score matching (*p* = 0.769)

Twenty nine of 55 patients in UL received neoadjuvant therapy and all patients in UL received adjuvant therapy after surgery. To clarify the influence of neoadjuvant therapy in UL, we compared DFS between UL cases that received and did not receive neoadjuvant therapy after propensity score matching to overcome heterogeneity between the two groups. Covariates included age, method of surgery, and pathological T stage (Table 3). There were 26 cases each that receive and did not receive neoadjuvant therapy, respectively, after propensity score matching. There was no significant difference in DFS between cases that received or did not receive neoadjuvant therapy (Fig. 2, *p* = 0.769).

**Table 3:** Propensity score matching description

Covariates	Total Population				Propensity-matched Population			
	non- NT (*n* = 26)	NT (*n* = 29)	p-value	standardized difference	non- NT (n = 26)	NT (n = 26)	p-value	standardized difference
Age	62.0 ± 8.0	59.5 ± 5.2	0.172	−0.479	62.0 ± 8.0	59.1 ± 5.3	0.130	−0.558
Ivor Lewis	23	22	0.303	0.289	23	22	0.500	0.088
pT Stage	2.4 ± 0.9	3.5 ± 0.5	< 0.001	2.084	2.4 ± 0.9	3.5 ± 0.5	< 0.001	2.193

## Discussion

Because clinical diagnosis of LN metastasis in esophageal squamous cell carcinoma is not exact and it is well known that LN metastasis is not often detected in small-sized lymph node (less than 10 mm), discrepancies between preoperative staging and pathologic findings are not uncommon, especially in locoregional LN [11]. Thoracic surgeons do not usually perform invasive evaluation for a locoregional LN when the LN is negative on PET-CT and upfront surgery without neoadjuvant therapy may be performed on patients with LN metastasis [9]. Although neoadjuvant therapy can increase the rate of postoperative complication or length of hospital stay for patients, neoadjuvant therapy followed by complete esophagectomy (R0) is well-established as being superior to upfront esophagectomy when LN metastasis is present. However, there are uncertainties regarding the management plan and prognosis for UL in esophageal cancer [2, 7, 14, 15]. In the present study, to clarify whether upfront surgery without neoadjuvant therapy in UL is appropriate, we compared prognosis between WL and UL and investigated the influence of neoadjuvant therapy in UL.

The present study showed that there was a substantial incidence of UL among preoperative cN0 patients and that a considerable incidence of UL did not receive neoadjuvant therapy. Therefore, the increased use of an invasive evaluation of a locoregional LN in esophageal cancer might be considered because neoadjuvant therapy is beneficial when LN metastasis is present. However, because there was no significant difference in DFS between WL and UL after upfront esophagectomy (R0) without neoadjuvant therapy, UL does not necessarily suggest the needs for increased use of invasive evaluation of a locoregional LN when the LN is determined to be negative by PET- CT.

The findings from the present study also suggested that continuing planned esophagectomy is appropriate if a surgeon faces an unsuspected LN during surgery in patients who will be able to tolerate adjuvant therapy after complete resection. In this study, all patients in UL received adjuvant therapy after surgery. The findings also suggest that surgeons do not need to routinely perform an intraoperative pathologic evaluation of a locoregional LN which was negative in preoperative evaluations to determine whether they continue a planned esophagectomy or not [18].

The present study showed that there was no significant benefit of neoadjuvant therapy in UL, consequently suggesting that upfront surgery without neoadjuvant therapy in UL is appropriate. However, one potential drawback to upfront surgery in UL is the possibility of surgical morbidity influencing the use of adjuvant therapy [19]. When surgeons face an unsuspected LN during surgery, they should consider the patient’s likely tolerability of esophagectomy and the subsequent adjuvant therapy [19, 20].

There are several limitations in the present study. First, the retrospective nature allows for the possibility of unobserved, and therefore uncontrolled, confounding factors or selection bias. Second, because of the small number of cases, especially in UL, and the possibility that the heterogeneity of the data could have affected the study findings, a propensity score matching method was carried out. Third, the shine-through phenomenon of PET-CT may also have affected preoperative evaluation.

## Conclusions

In the present study, a propensity matched analysis showed that there were no significant differences in DFS between WL and UL after complete resections. In addition, neoadjuvant therapy did not confer a survival benefit in UL when adjuvant therapy was performed after complete resection. Therefore, preoperative invasive evaluation for a locoregional LN is not necessary when the LN is negative on PET-CT and upfront surgery without neoadjuvant therapy in UL is appropriate for patients that will be able to tolerate adjuvant therapy.

## Abbreviations

LN: Lymph node
UL: Patients with unsuspected lymph node metastasis
WL: Patients without lymph node metastasis
